# Neuroadaptive changes in brain structural–functional coupling among pilots

**DOI:** 10.3389/fnins.2025.1608739

**Published:** 2025-07-24

**Authors:** Xi Chen, Qingbin Meng, Qingsong Song, Peiran Xu, Shicong Zhang, Donglin Huang, Qi Chu, Jiamin Fan, Cheng Luo, Xiuyi Li

**Affiliations:** 1 Institute of Flight Technology, Civil Aviation Flight University of China, Guanghan, Sichuan, China; 2 Aviation Health Department, Southwest Regional Administration of Civil Aviation Administration of China, Chengdu, China; 3 Hospital of Civil Aviation Flight University of China, Civil Aviation Flight University of China, Guanghan, Sichuan, China; 4 Key Laboratory for Neuroinformation of Ministry of Education, School of Life Sciences and Technology, University of Electronic Science and Technology of China, Chengdu, China; 5 CAAC Academy, Civil Aviation Flight University of China, Guanghan, China

**Keywords:** structural-functional coupling, flying experience, neuroplasticity, function connectivity, graph signal processing

## Abstract

**Background:**

Investigating the neural mechanisms underlying pilots’ brains is crucial for enhancing aviation safety. However, prior research has predominantly focused on identifying structural and functional differences in the brain, while the relationship between structure and function remains insufficiently elucidated.

**Methods:**

This study collected T1-weighted structural magnetic resonance imaging (MRI), resting-state functional MRI (rs-fMRI), and diffusion tensor imaging (DTI) data from 47 pilots and 38 matched controls. Structural–functional coupling (SFC) strength was quantified using the Structural Decoupling Index (SDI) based on graph signal processing (GSP). Functional connectivity was further decomposed into structurally coupled and decoupled components, with subsequent group comparisons conducted at the regional brain level.

**Results:**

Compared to controls, pilots exhibited significantly higher SDI values in several brain regions, including the left and right middle frontal gyri, left precentral gyrus, inferior temporal gyrus, left posterior superior temporal sulcus, right superior and inferior parietal lobules, left visual cortex, and right basal ganglia, indicating reduced SFC in these areas. In contrast, enhanced coupling was observed in the bilateral inferior frontal gyri, left paracentral lobule, and left insula. Notably, pilots showed increased decoupled functional connectivity (d-FC) between the left cuneus and right insula, as well as between the right insula and the left medial occipital cortex, accompanied by a reduction in coupled functional connectivity (c-FC). Importantly, the strength of decoupled functional connectivity between the right insula and the left medial occipital cortex was positively correlated with total flight hours.

**Conclusion:**

These findings suggest that prolonged flight experience may induce neuroplastic changes in regional SFC within the brains of pilots. This work provides novel insights into the neural adaptations associated with flight training and may contribute to the refinement of pilot selection and training protocols aimed at improving aviation safety.

## 1 Introduction

Pilots are professionals who operate under high levels of stress and must respond rapidly to constantly changing flight environments. In recent years, the proportion of aviation accidents attributed to human factors has increased significantly (Wang H. et al., 2020). Although extensive research has been dedicated to reducing human errors caused by pilots’ psychological traits, such as hazardous attitudes and risk tolerance (Ji et al., 2011; Lee and Park, 2016), as well as cognitive deficiencies, including limited working memory, improper attention allocation, and spatial disorientation (Bałaj et al., 2019; Seyfzadehdarabad et al., 2023), human factors remain a critical contributor to compromised flight safety (Kharoufah et al., 2018). Therefore, in-depth research on the neural mechanisms underlying pilot brain function is essential to improve flight safety.

Magnetic resonance imaging (MRI) has recently emerged as an indispensable tool for exploring brain structure and functional plasticity due to the modality’s non-invasive nature and high spatial resolution. Neuroplasticity has demonstrated that skill acquisition and experiential learning can induce plastic changes in both brain structure and function (Chen et al., 2006; Bezzola et al., 2011; May, 2011). For instance, functional imaging studies have demonstrated that pilots display distinct activation patterns during sensorimotor tasks, especially in regions related to observational learning and motor simulation, differentiating them from non-pilots (Callan et al., 2013). Furthermore, flight training can increase the degree of centrality in the prefrontal and occipital cortices, potentially improving executive functions (Chen et al., 2023). Studies have observed structural changes, indicating that airline pilots have greater gray and white matter volumes in the visual, sensorimotor, and prefrontal parietal regions compared to the general population (Qiu et al., 2021). Our previous research also suggests that flight training can increase the gray matter volume (GMV) of the lingual and fusiform gyri (Xu et al., 2023). Although these findings highlight significant brain changes in pilots, current methods are still not fully elucidating the influence of anatomical constraints on functional brain activity.

Understanding the structural constraints of functional brain activity is a significant area of research in cognitive neuroscience (Stiso and Bassett, 2018). Structural–functional coupling (SFC) reflects the extent to which structural connectivity (SC) supports functional connectivity (Baum et al., 2020). A deep understanding of SFC is essential to clarify how white matter structures promote sensory integration and executive functions (Rajesh et al., 2024). Early studies typically employed simple correlation analyses to explore this relationship (Honey et al., 2009; Mišić et al., 2016; Amico and Goñi, 2018). However, as the field has advanced, more complex approaches, such as communication and biophysical models, have emerged (Deco et al., 2011; Cabral et al., 2017; Mišić et al., 2018; Seguin et al., 2018). Recently, graph signal processing (GSP) has provided a novel framework for analyzing brain imaging data (Huang et al., 2018). GSP-based methods introduce a Structural Decoupling Index (SDI) to reflect the alignment of each brain region with its SC. This approach projects functional signals onto the structural harmonic space via the eigendecomposition of SC, decomposing graph signals into low-frequency and high-frequency components. The ratio of the energies of these components constitutes the SDI (Preti and Van De Ville, 2019). Existing studies have used GSP to investigate how dance and musical training affect the SFC in the brains of dancers and musicians (Gao et al., 2024).

To assess the influence of flight experience on structural–functional relationships in the brains of pilots, we recruited a group of professional pilots with extensive flight experience and a matched control group comprising individuals from various occupational backgrounds, who had no prior flight experience. The two groups were carefully matched for age, gender, and educational background. First, we applied GSP to examine the coupling between functional and structural signals at the regional level. Next, we explored how the coupling and decoupling components of functional signals contribute to interregional functional connectivity. Finally, we evaluated whether these metrics were related to the training experience. This study aimed to explore the neural mechanisms of pilots’ brains to provide a scientific basis for optimizing pilot training and selection programs.

## 2 Materials and methods

### 2.1 Methods outline

**FIGURE 1:**
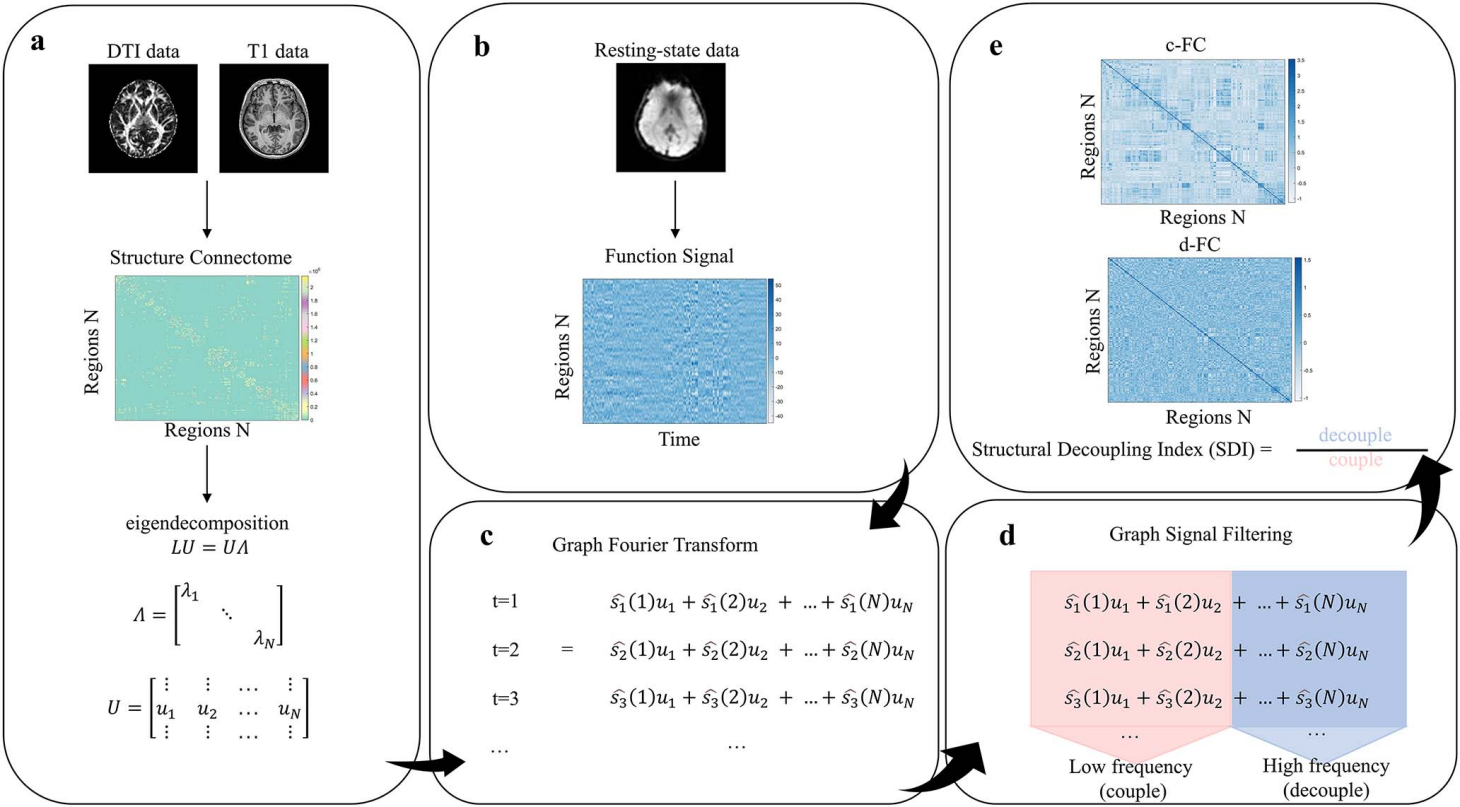
Methods pipeline **(a)** Construct structural connectivity matrix and perform eigen-decomposition. **(b)** Construct functional signal matrix. **(c)** Graph Fourier Transform (GFT). **(d)** Decompose functional signals via graph signal filtering. **(e)** Calculate structural decoupling index (SDI) and construct coupled/decoupled functional connectivity matrices.

We first collected T1-weighted structural images, resting-state functional MRI (rs-fMRI), and diffusion tensor imaging (DTI), followed by preprocessing procedures. SFC strength was quantified using the SDI, within the framework of GSP (Preti and Van De Ville, 2019). Specifically, a graph Laplacian matrix was constructed to represent the brain’s structural network, and eigendecomposition was performed to obtain eigenvectors serving as harmonic components of the connectome. Functional signals were then decomposed into coupled and decoupled components via the Graph Fourier transform (GFT). SDI was defined as the log-ratio of the energy in the decoupled to coupled components (Figure 1).

### 2.2 Participants

This study was approved by the Ethics Committee of the University of Electronic Science and Technology of China (Chengdu) (Approval Number: 1420200408-07) and conducted in strict accordance with the principles outlined in the 1964 Declaration of Helsinki. Prior to participating in the experiment, all participants signed an informed consent form. The pilot group consisted of 47 experienced male airline pilots with flight hours ranging from 350 to 19,000. The control group comprised 38 flight-naïve individuals from various occupational backgrounds, matched to the pilot group by age, gender, handedness, and years of education. Exclusion criteria included: (1) claustrophobia, (2) history of psychiatric or neurological disorders, and (3) substance dependence.

### 2.3 MRI data acquisition

All imaging was performed on a 3T MRI scanner at the University of Electronic Science and Technology of China. T1-weighted images were acquired using a three-dimensional (3D) fast spoiled gradient recalled echo sequence. The parameters were as follows: 156 slices, TR = 6.012 ms, TE = 1.9872 ms, FOV = 256 mm × 256 mm, FA = 9°, matrix = 256 × 256, slice thickness = 1 mm. DTI data were acquired using a diffusion-weighted spin echo EPI sequence with the following parameters: TR = 8500 ms, TE = 67 ms, FOV = 256 mm × 256 mm, matrix = 128 × 128, slice thickness = 2 mm, 78 slices, *b* = 1000 s/mm. rs-fMRI data were acquired using a gradient echo planar imaging (EPI) sequence with an eight-channel phased-array head coil. The scanning parameters were as follows: TR = 2000 ms, TE = 30 ms, FA = 90°, matrix = 64 × 64, FOV = 240 mm × 240 mm, slice thickness = 4 mm.

During scanning, foam pads were used to minimize head motion. Participants were instructed to keep their eyes closed, remain relaxed, stay awake, and refrain from engaging in deliberate thinking.

### 2.4 MRI data preprocessing

Functional MRI data were preprocessed using the SPM12 toolbox in MATLAB R2018b. To allow for signal stabilization, the first five volumes were discarded. The remaining 250 volumes were subjected to slice timing correction and realignment for head motion correction. To minimize potential artifacts caused by head motion, participants with head motion exceeding 2.5 mm of translation or 2.5° of rotation were excluded to reduce motion-related artifacts. Subsequently, functional images were coregistered with corresponding T1-weighted structural images, normalized to the Montreal Neurological Institute (MNI) space (voxel size = 3 mm × 3 mm × 3 mm), and spatially smoothed using an 8 mm full width at half maximum (FWHM) Gaussian kernel. Additionally, nuisance regression was performed to remove confounding signals, including Friston-24 motion parameters, whole-brain mean, white matter, and cerebrospinal fluid (CSF) signals. Finally, linear detrending and band-pass filtering (0.01–0.08 Hz) were applied.

Diffusion MRI data were preprocessed using FSL. First, non-brain tissues were removed from the b0 image using the Brain Extraction Tool (BET) to generate a brain mask. Head motion and eddy current distortions were corrected using the eddy tool. Diffusion tensors were estimated using DTIFIT to compute scalar maps such as fractional anisotropy (FA). Voxel-wise fiber orientation distributions were then modeled using BEDPOSTX, which applies Markov Chain Monte Carlo (MCMC) sampling to account for crossing fibers. Finally, probabilistic tractography was performed using PROBTRACKX2, with tracking parameters set to 5,000 samples per seed voxel, a step length of 0.5 mm, and a curvature threshold of 0.2.

T1-weighted images were preprocessed using the Computational Anatomy Toolbox (CAT12.7, r1742) implemented in SPM12, running on MATLAB R2018b. Images were first reoriented to align with the anterior commissure–posterior commissure (AC–PC) plane, followed by bias field correction. The images were then segmented into gray matter (GM), white matter (WM), and CSF. Subsequently, the segmented images were normalized to MNI space (voxel size = 1.5 mm × 1.5 mm × 1.5 mm), modulated to preserve volume information, and smoothed using a 4 mm FWHM Gaussian kernel.

### 2.5 Structural connectivity matrix and functional signal matrix

The brain was first parcellated into 246 regions based on the Human Brainnetome Atlas, which comprises 210 cortical regions and 36 subcortical regions (Fan et al., 2016). Then, white matter fiber connectivity between the 246 brain regions defined by the Human Brainnetome Atlas was estimated using the PROBTRACKX2 tool in FSL. Subsequently, the GMV of each region was extracted from the T1-weighted images. The number of fiber tracts between any two regions was normalized by the sum of their GMVs to produce a 246 × 246 structural connectivity matrix for each participant. Finally, the mean BOLD signal within each brain region was calculated by averaging across all voxels within the region, resulting in a 246 × 250 functional signal matrix, where 250 refers to the number of time points remaining after removing the first five volumes of rs-fMRI data.

### 2.6 Structural connectivity harmonics and GFT

First, the SC matrix was defined as an adjacency matrix *A*. A symmetric normalized graph Laplacian matrix *L* was then computed as *L* = *I*−*D*^−1/2^*AD*^−1/2^, where *D* is the degree matrix, and *I* is the identity matrix. The matrix *L*characterizes the topological structure and connectivity between brain regions. Eigendecomposition was applied to obtain the eigenvectors and eigenvalues of *L*, denoted as *LU* = *U*Λ, where *U* contains the eigenvectors and Λ is a diagonal matrix of eigenvalues. The eigenvalues λ_*k*_ are interpreted as frequencies, and the corresponding eigenvectors *u*_*k*_ serve as the SC harmonics. Eigenvectors associated with low-frequency eigenvalues represent smooth variations over the network structure. Functional signals were then projected into the spectral domain using the GFT: ${\hat{s}}_{t}=U^{T}\,s_{t}$.

### 2.7 Measurement of SDI

Graph signal filtering was applied to decompose the functional signal into two components: one that aligns closely with the brain’s structural topology (coupled), and one that diverges from it (decoupled). The cut-off frequency *C* was determined to divide the average spectral energy density into two equal parts. The coupled and decoupled signals were obtained using the following equations: $s_{t}^{C}=U_{\mathrm{low}}\,U^{T}\,s_{t}$ and $s_{t}^{D}=U_{\mathrm{high}}\,U^{T}\,s_{t}$, where *U*_low_ *and* *U*_high_ contain the eigenvectors associated with the low and high frequencies, respectively. These filtered signals $s_{t}^{C}\,\mathrm{and}\,s_{t}^{D}$ structure-aligned and structure-independent components of the functional signal. For each brain region, the SDI was defined as the base-2 logarithmic ratio of the L2 norms of the decoupled and coupled components: $\mathrm{SDI}=\log_{2}\,\frac{∥s_{t}^{D}∥}{∥s_{t}^{C}∥}$ . A negative SDI indicates strong alignment (coupling) between structure and function in that region, while a positive SDI suggests relative independence (decoupling). Additionally, we calculated the Pearson’s correlation coefficients between $s_{t}^{C}\,\mathrm{and}\,s_{t}^{D}$ across regions, resulting in two 246 × 246 functional connectivity matrices: the coupled functional connectivity (c-FC) matrix and the decoupled functional connectivity (d-FC) matrix. c-FC refers to components of functional connectivity that are highly consistent with SC, reflecting functional activities that rely on stable anatomical pathways; whereas d-FC refers to components of functional connectivity that are independent of SC, potentially reflecting greater flexibility or adaptability (Griffa et al., 2022).

### 2.8 Statistical analysis

Two-sample *t*-tests were first conducted to compare regional differences in SDI between the pilot and control groups. Statistical significance was assessed using a non-parametric permutation test with 5,000 iterations. A two-tailed test was applied, and the significance threshold was set at *p* < 0.05. Subsequently, Fisher’s Z-transformation was applied to the c-FC and d-FC matrices, followed by a 2 × 2 repeated-measures analysis of variance (ANOVA). To control for multiple comparisons, family-wise error (FWE) correction was applied, with the significance threshold set at *p* < 0.01. Age, years of education, total intracranial volume (TIV), and mean framewise displacement (mFD) were included as covariates in all statistical analyses. Finally, the effects of age, years of education, TIV, and mFD were regressed out from the data, and outliers were subsequently removed from the residuals. Spearman’s correlation analysis was then performed to examine the relationship between coupling and decoupling functional connectivity among the brain regions showing significant interaction effects in the repeated-measures ANOVA and total flight hours.

## 3 Results

### 3.1 Participant demographic information

A total of 47 male airline pilots (mean age: 34.3 ± 5.4 years; mean education: 16.1 ± 0.97 years) and 38 male flight-naïve controls (mean age: 34.0 ± 4.1 years; mean education: 16.3 ± 1.1 years) were included in the analysis (Table 1). Independent samples *t*-tests revealed no significant group differences in age (*t* = 0.656, *p* = 0.514) or years of education (*t* = −0.057, *p* = 0.955).

**TABLE 1:** Basic information of the two groups.

Characteristics	Pilot (*n* = 47)	Control (*n* = 38)	*t*-value	*P*-value
Sex (male)	47	38		
Age (yr)	34.3 (5.4)	34 (4.1)	0.656	0.514^a^
Education years	16.1 (0.97)	16.3 (1.1)	−0.057	0.955^a^

^a^Represents the independent samples *t*-test. Values are presented as mean (standard deviation).

### 3.2 Whole-brain distribution of structural–functional coupling

**FIGURE 2:**
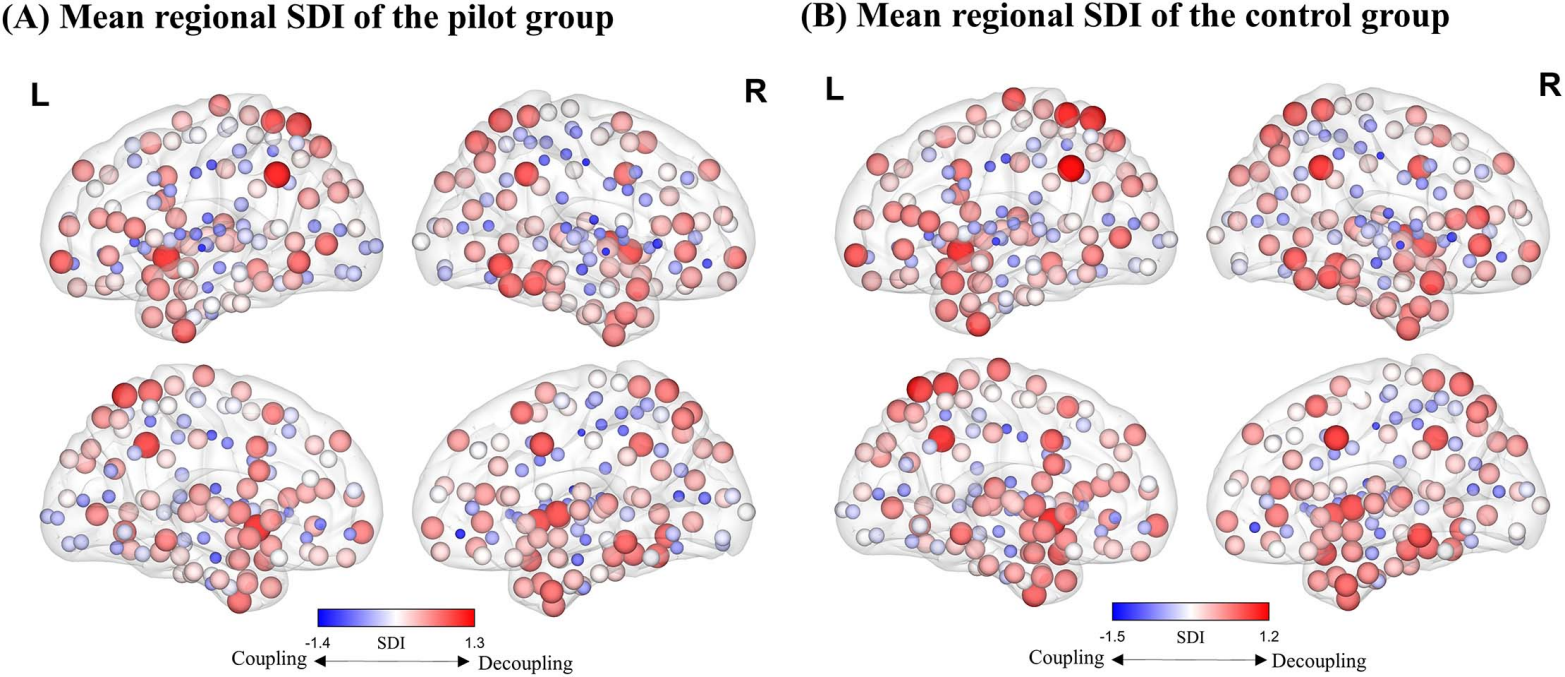
Distribution map of the regional average Structure-Decoupling Index (SDI). **(A)** Average SDI map for the pilot group. **(B)** Average SDI map for the control group. Node size is proportional to SDI values, and node color indicates SDI magnitude. Larger positive SDI values indicate greater decoupling, while smaller negative SDI values reflect stronger coupling.

As illustrated in Figure 2, our findings reveal a hierarchical distribution pattern consistent with previous research. In unimodal cortex (including primary sensory and motor regions), SFC is generally higher (i.e., more negative SDI values), indicating that functional activity in these areas is heavily reliant on stable structural connections. In contrast, transmodal cortex exhibits lower levels of SFC (i.e., more positive SDI values).

### 3.3 Group differences in regional structural-functional coupling

**FIGURE 3:**
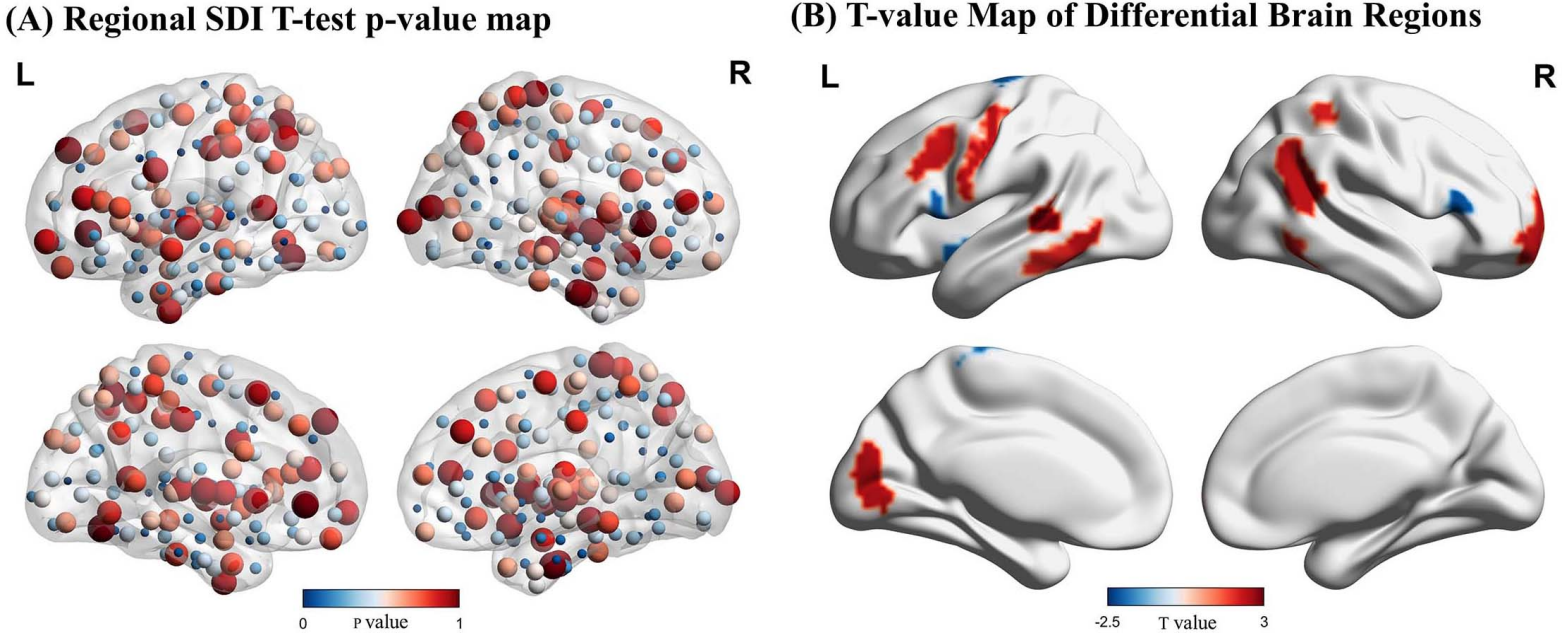
Results of the Structural Decoupling Index (SDI) difference analysis. **(A)** Regional SDI differences represented by node color based on *p*-values from *t*-tests. Larger nodes correspond to larger *p*-values. **(B)** *T*-value map illustrating brain regions with significant SDI differences. Positive *t*-values (red) indicate regions with greater decoupling (higher SDI values) in pilots, while negative *t*-values (blue) represent regions with greater coupling (lower SDI values) relative to controls. GFT, Graph Fourier Transform.

Two-sample *t*-tests identified significant differences in SDI across 16 regions of the brain (*p* < 0.05). Compared to controls, pilots exhibited significantly higher SDI values in the left and right middle frontal gyri, left precentral gyrus, inferior temporal gyrus, left posterior superior temporal sulcus, right superior and inferior parietal lobules, left visual cortex, and right basal ganglia, indicating reduced SFC in these areas. In contrast, increased coupling was observed in the bilateral inferior frontal gyri, left paracentral lobule, and left insula in the pilot group (Figure 3 and Supplementary Table 1).

### 3.4 Interaction effects in functional connectivity

**FIGURE 4:**
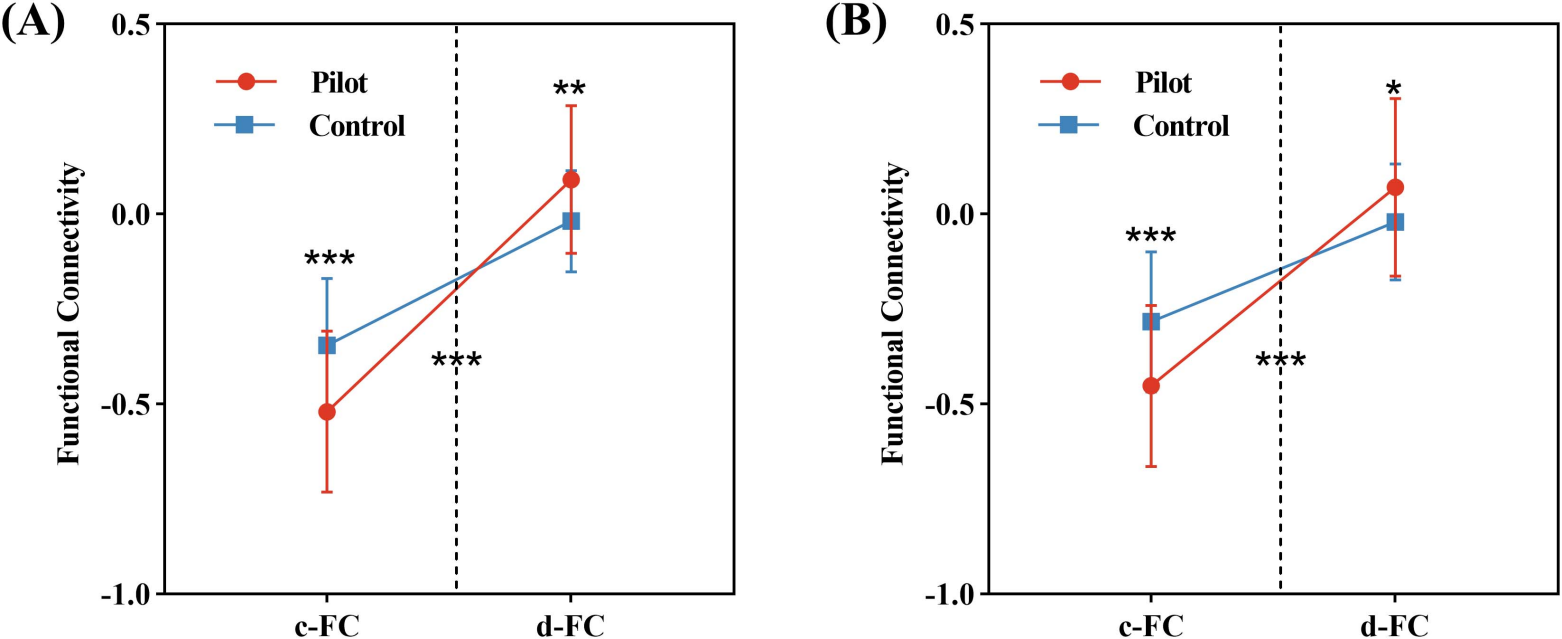
Results of the repeated-measures ANOVA. **(A)** Significant interaction effect between FC components (c-FC and d-FC) and group in connectivity between the left cuneus and the right insula. **(B)** Significant interaction effect for connectivity between the right insula and the left medial occipital cortex. Statistical significance indicated as: *P < 0.05, **P < 0.01, ***P < 0.001 (FWE-corrected *P* < 0.01).

Repeated-measures ANOVA revealed significant interaction effects in functional connectivity between the left cuneus and right insula, as well as between the right insula and left medial occipital cortex (FWE-corrected *p* < 0.01). Simple effects analysis showed that c-FC between these regions was significantly reduced, while decoupled connectivity was increased (Figure 4).

### 3.5 Correlation of d-FC with behavioral measures

**FIGURE 5:**
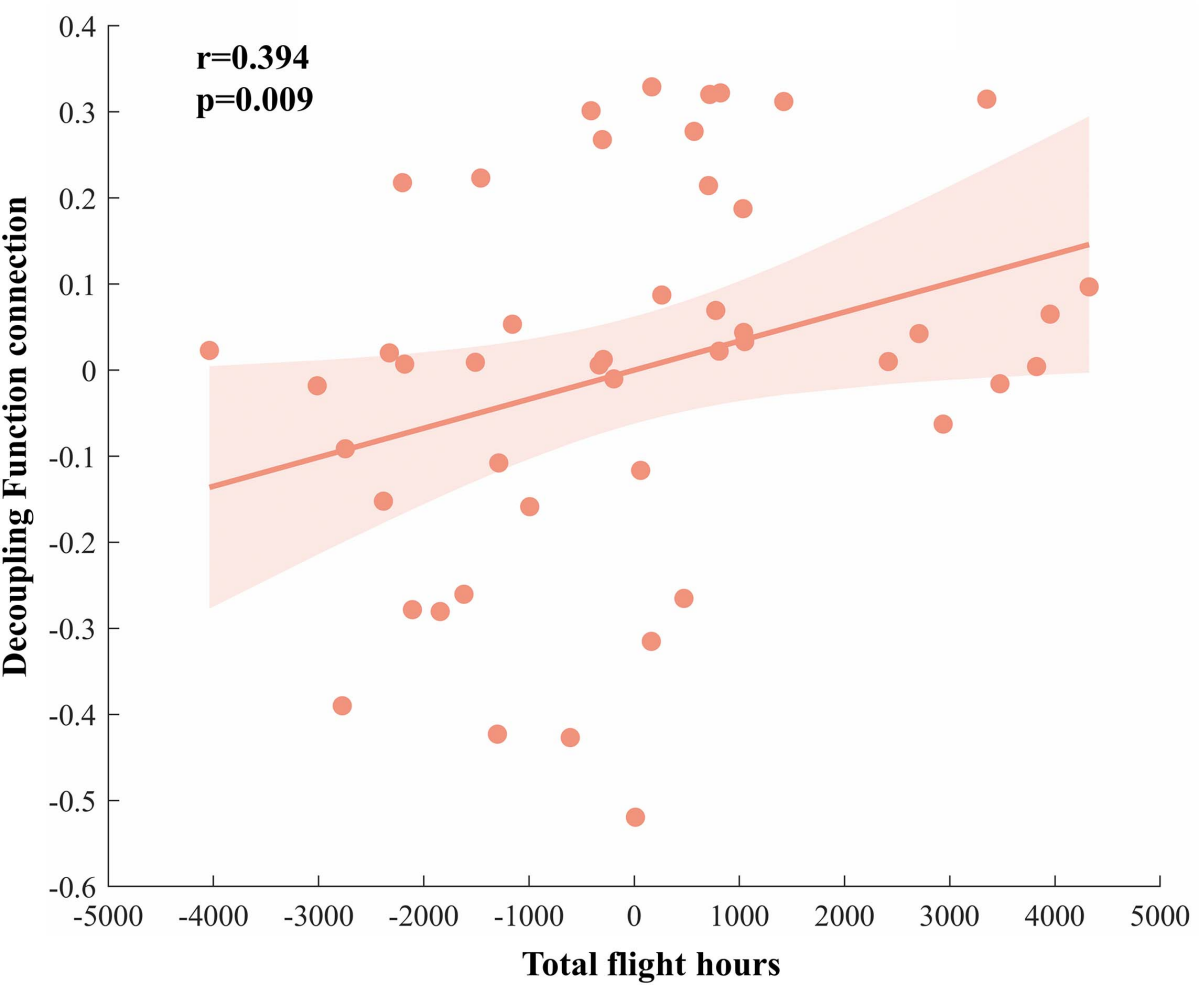
Correlation analysis results. The decoupling functional connectivity between the right insula and left medial occipital cortex in the pilot group is positively correlated with total flight hours.

We examined the correlation between functional connectivity and flight experience. Spearman’s correlation analysis revealed a significant positive relationship between d-FC of the right insula and the left medial occipital cortex and total flight hours (r^2^ = 0.155, *p* = 0.009; Figure 5).

## 4 Discussion

This study employed a GSP framework to investigate differences in SFC between airline pilots and matched controls, aiming to elucidate neuroplastic changes associated with long-term flight experience. The results revealed altered SFC patterns in several brain regions among pilots. Specifically, reduced coupling was observed in the bilateral middle frontal gyri, inferior temporal gyri, right inferior and superior parietal lobules, and left visual cortex. In contrast, increased coupling was identified in the bilateral inferior frontal gyri, left paracentral lobule, and left insula. Furthermore, pilots demonstrated significantly increased d-FC between the left cuneus and right insula, as well as between the right insula and the left medial occipital cortex, accompanied by a reduction in c-FC. Notably, the strength of d-FC between the right insula and the left medial occipital cortex was positively correlated with total flight hours, suggesting experience-dependent modulation of structure-independent functional interactions.

This study found that both pilots and controls exhibited a hierarchical distribution of whole-brain SFC, consistent with previous findings. Notably, the pilot group demonstrated significantly reduced SFC in several transmodal association cortical regions, including the middle frontal gyrus, inferior temporal gyrus, and both the superior and inferior parietal lobules. Previous neuroimaging studies have highlighted the importance of reduced SFC in transmodal association cortices for cognitive flexibility and adaptability (Vázquez-Rodríguez et al., 2019). The parietal cortex is considered a “flexible hub” for multitask control, playing a key role in task switching and integration under complex environmental conditions (Cole et al., 2013). The inferior temporal gyrus is primarily associated with complex visual representations and semantic processing, forming a critical basis for high-level cognitive functions (Lin et al., 2020). Prior research suggests that cognitive flexibility depends on the multimodal integration capacity of prefrontal and parietal regions (Uddin, 2021). In general, reduced SFC implies that functional activity is less constrained by anatomical architecture, which may reflect enhanced flexibility and adaptability (Baum et al., 2020). Moreover, a healthy brain can dynamically adjust its level of flexibility in response to changing environmental demands (Egner and Siqi-Liu, 2024). Based on these findings and prior literature, we speculate that the reduced SFC observed in these regions among pilots may reflect neuroadaptive remodeling driven by long-term engagement in complex flight tasks. These changes may support superior cognitive flexibility and help reduce operational risk. This hypothesis is consistent with prior studies linking lower SFC to greater cognitive flexibility and more effective task performance (Wu et al., 2020; Rajesh et al., 2024). Our earlier research also found that pilots exhibited increased inter-network connectivity, decreased intra-network connectivity, and increased brain-state transition frequency, which may underlie their cognitive flexibility (Chen et al., 2020). Moreover, a recent study based on the triple-network model demonstrated enhanced inter-network connectivity in pilot trainees during dynamic functional connectivity analysis, suggesting superior cognitive flexibility in managing complex tasks (Ye et al., 2025). However, it should be noted that direct behavioral evidence supporting these neural mechanisms is still lacking. Future studies should incorporate task paradigms to further elucidate the functional implications and behavioral correlates of altered SFC in pilots.

Moreover, we observed increased SFC in the inferior frontal gyrus (IFG) among pilots. Neuroimaging evidence suggests that the IFG plays a critical role in various cognitive processes, including language processing, attentional capture, and response inhibition (Wang J. et al., 2020; Rivas-Fernández et al., 2021; Wang, 2025). It is also implicated in multisensory integration, particularly exhibiting significant integrative functions during category learning tasks (Li et al., 2020). From a neurobiological perspective, enhanced SFC is generally regarded as a marker of increased neural pathway stability and regional functional specialization (Baum et al., 2020). A previous study on fighter pilots found reduced functional connectivity strength of the IFG within whole-brain networks compared to the general population, suggesting a potentially higher degree of specialized processing in this region (Radstake et al., 2023). Therefore, we speculate that the increased SFC in the IFG may reflect the development of more stable and specialized neural pathways resulting from long-term engagement in complex perception and action environments, thereby supporting rapid detection and efficient regulation of linguistic cues and attentional resources during high-demand task execution. It is worth noting, however, that there remains debate as to whether the IFG is specifically involved in semantic and language processing or whether it serves broader domain-general cognitive functions (Yeo et al., 2015). Accordingly, future studies should incorporate well-designed behavioral paradigms and cognitive assessments to further elucidate the functional significance of the observed increase in coupling—namely, whether it represents enhanced functional specialization or a general improvement in cognitive capacity.

For pilots, spatial navigation is a highly complex cognitive task that requires continuous monitoring of flight instruments and directional judgment, particularly in the absence of external postural or motion cues (Dahlstrom and Nahlinder, 2009; Bałaj et al., 2019). Previous studies have shown that pilots exhibit significant activation in the frontal and parietal cortices during stressful situations, observational learning tasks, and spatial cognition assessments (Sladky et al., 2016; Causse et al., 2019, 2024). As a key region involved in higher-order cognitive processes, the prefrontal cortex plays a critical role in spatial working memory and spatial attention tasks (Funahashi, 2017). The inferior parietal lobule is involved in the integration of sensory information, spatial localization, and sustained attention (Wang et al., 2001; Salgado-Pineda et al., 2003; Behrmann et al., 2004; Buchsbaum et al., 2005), while the superior parietal lobule primarily integrates auditory and visual inputs and supports goal-directed spatial orienting (Shomstein et al., 2010). These two regions operate in close coordination during spatial attention and orienting tasks, performing essential functions (Vandenberghe et al., 2012). In the present study, we observed significantly reduced SFC in the middle frontal gyrus, superior parietal lobule, and inferior parietal lobule in the pilot group. According to existing literature, weakened SFC in specific brain regions may be associated with enhanced executive function and memory performance (Griffa et al., 2022). Furthermore, flight experience has been shown to improve cognitive capacity and mitigate the effects of cognitive aging (Causse et al., 2011). Based on these findings, we speculate that pilots undergo specific neuroadaptive remodeling processes as a result of prolonged engagement in cognitively demanding tasks. These neural changes may enhance executive control and memory functions, thereby reducing operational errors and improving aviation safety. This interpretation is also consistent with our previous findings. We previously demonstrated increased degree centrality in the left middle frontal gyrus following flight training, and this change was significantly associated with better executive function performance (Chen et al., 2023).

The insula is a core brain region responsible for multimodal information integration, with widespread connections to various cortical and subcortical areas. It plays a vital role in integrating sensory, affective, and cognitive information (Evrard, 2019). In addition to its involvement in spatial orientation, the insula also contributes to higher-order spatial navigation processes (Qiu et al., 2019; Baier et al., 2021; Bleau et al., 2022). The precuneus is critical for spatial orientation and visual task processing (Grill-Spector and Malach, 2004; Parker et al., 2014). Recent studies have identified the precuneus as a key connectivity hub, characterized by dense temporo-occipital and extensive long-range connections (Palejwala, 2021), further underscoring its role in visual information processing. Increased white matter volume in the precuneus among pilots has been reported, which may reflect the high demand for visual processing during flight tasks (Qiu et al., 2021). In addition, the middle occipital gyrus (MOG) is involved in higher-level visual processing and plays an important role in rapid decision-making and task execution (Aberbach-Goodman and Mukamel, 2023; Wang et al., 2024). Coordinated activity among these regions provides essential neural support for pilots operating under high-demand conditions, particularly in spatial orientation and visual processing. In the present study, we found that pilots exhibited enhanced d-FC between the left insula and the left cuneus, as well as between the left insula and the left MOG. The functional decoupling is thought to support an individual’s capacity to integrate novel information, and previous studies have shown that decoupled connectivity is closely related to sustained attention (Griffa et al., 2022). Further analysis revealed that the degree of functional decoupling between the insula and the MOG was positively correlated with total flight hours among pilots. This finding suggests that long-term flight experience may induce adaptive remodeling of the connectivity patterns between the insula and visual cortices, thereby enhancing pilots’ performance during complex flight operations. Our previous research also demonstrated that flight training enhances functional connectivity between parietal and occipital cortices in pilot trainees (Chen et al., 2024). In addition, other studies have indicated that exposure to different gravity environments can significantly affect functional brain networks. For instance, short-term high-G acceleration experienced frequently by fighter pilots (Radstake et al., 2023) and prolonged microgravity exposure in astronauts (Jillings et al., 2023) have both been associated with extensive reorganization of functional brain networks. The present findings extend this line of research, jointly supporting the notion that flight experience and exposure to extreme environments exert profound effects on large-scale brain functional architecture.

## 5 Limitation

This study has several limitations: (1) We did not control for the type of education received by participants. Although most individuals in the control group were employed in science- and engineering-related fields, variations in educational content and training approaches may still influence brain function. Therefore, future studies should carefully control for this factor. (2) The use of resting-state fMRI in this study may underestimate brain network reorganization during actual task execution in pilots. Future research should incorporate task-based fMRI or flight simulation paradigms to more comprehensively elucidate the underlying functional mechanisms. (3) This study lacks a longitudinal design, thus limiting our ability to establish causality. Future research should adopt a longitudinal approach to better elucidate how flight experience impacts the brain’s structure-function relationships. (4) This study did not include direct assessments of cognitive function in pilots. Including such measures, particularly those assessing cognitive flexibility, would help clarify the behavioral relevance of the observed neural differences and provide further insight into their functional implications.

## 6 Conclusion

In conclusion, this study investigated the structural-functional coupling of the pilot’s brains within a novel research framework. We noted that prolonged flight experience can induce changes in the structural-functional coupling strength, thus enhancing our understanding of the effects of flight on neural plasticity. The results may provide insights for optimizing future pilot selection and training programs. However, further research is needed to validate these findings.

## Data Availability

The datasets presented in this study can be found in online repositories. The names of the repository/repositories and accession number(s) can be found below: https://osf.io/xhqdk/.

## References

[B1] Aberbach-Goodman S. Mukamel R. (2023). Temporal hierarchy of observed goal-directed actions. *Sci. Rep.* 13:19701. 10.1038/s41598-023-46917-z 37952024 PMC10640622

[B2] Amico E. Goñi J. (2018). Mapping hybrid functional-structural connectivity traits in the human connectome. *Netw. Neurosci.* 2 306–322. 10.1162/netn_a_00049 30259007 PMC6145853

[B3] Baier B. Cuvenhaus H. S. Müller N. Birklein F. Dieterich M. (2021). The importance of the insular cortex for vestibular and spatial syndromes. *Eur. J. Neurol.* 28 1774–1778. 10.1111/ene.14660 33270346

[B4] Bałaj B. Lewkowicz R. Francuz P. Augustynowicz P. Fudali-Czyż A. Stróżak P. (2019). Spatial disorientation cue effects on gaze behaviour in pilots and non-pilots. *Cogn. Tech. Work* 21 473–486. 10.1007/s10111-018-0534-7

[B5] Baum G. L. Cui Z. Roalf D. R. Ciric R. Betzel R. F. Larsen B. (2020). Development of structure–function coupling in human brain networks during youth. *Proc. Natl. Acad. Sci. U.S.A.* 117 771–778. 10.1073/pnas.1912034117 31874926 PMC6955327

[B6] Behrmann M. Geng J. J. Shomstein S. (2004). Parietal cortex and attention. *Curr. Opin. Neurobiol.* 14 212–217. 10.1016/j.conb.2004.03.012 15082327

[B7] Bezzola L. Mérillat S. Gaser C. Jäncke L. (2011). Training-induced neural plasticity in golf novices. *J. Neurosci.* 31 12444–12448. 10.1523/JNEUROSCI.1996-11.2011 21880905 PMC6703254

[B8] Bleau M. Paré S. Chebat D.-R. Kupers R. Nemargut J. P. Ptito M. (2022). Neural substrates of spatial processing and navigation in blindness: An activation likelihood estimation meta-analysis. *Front. Neurosci.* 16:1010354. 10.3389/fnins.2022.1010354 36340755 PMC9630591

[B9] Buchsbaum B. R. Greer S. Chang W. Berman K. F. (2005). Meta-analysis of neuroimaging studies of the Wisconsin Card-Sorting task and component processes. *Hum. Brain Mapp.* 25 35–45. 10.1002/hbm.20128 15846821 PMC6871753

[B10] Cabral J. Kringelbach M. L. Deco G. (2017). Functional connectivity dynamically evolves on multiple time-scales over a static structural connectome: Models and mechanisms. *Neuroimage* 160 84–96. 10.1016/j.neuroimage.2017.03.045 28343985

[B11] Callan D. E. Terzibas C. Cassel D. B. Callan A. Kawato M. Sato M. (2013). Differential activation of brain regions involved with error-feedback and imitation based motor simulation when observing self and an expert’s actions in pilots and non-pilots on a complex glider landing task. *Neuroimage* 72 55–68. 10.1016/j.neuroimage.2013.01.028 23357079

[B12] Causse M. Chua Z. K. Rémy F. (2019). Influences of age, mental workload, and flight experience on cognitive performance and prefrontal activity in private pilots: A fNIRS study. *Sci. Rep.* 9:7688. 10.1038/s41598-019-44082-w 31118436 PMC6531547

[B13] Causse M. Dehais F. Arexis M. Pastor J. (2011). Cognitive aging and flight performances in general aviation pilots. *Aging Neuropsychol. Cogn.* 18 544–561. 10.1080/13825585.2011.586018 21819276

[B14] Causse M. Mouratille D. Rouillard Y. El Yagoubi R. Matton N. Hidalgo-Muñoz A. (2024). How a pilot’s brain copes with stress and mental load? Insights from the executive control network. *Behav. Brain Res.* 456:114698. 10.1016/j.bbr.2023.114698 37797721

[B15] Chen F. Hu Z. Zhao X. Wang R. Yang Z. Wang X. (2006). Neural correlates of serial abacus mental calculation in children: A functional MRI study. *Neurosci. Lett.* 403 46–51. 10.1016/j.neulet.2006.04.041 16697526

[B16] Chen X. Jiang H. Meng Y. Xu Z. Luo C. (2024). Increased Functional connectivity between the parietal and occipital modules among flight cadets. *Aeros. Med. Hum. Perform.* 95 375–380. 10.3357/AMHP.6370.2024 38915163

[B17] Chen X. Wang Q. Luo C. Yang Y. Jiang H. Guo X. (2020). Increased functional dynamics in civil aviation pilots: Evidence from a neuroimaging study. *PLoS One* 15:e0234790. 10.1371/journal.pone.0234790 32555721 PMC7302522

[B18] Chen X. Wang Z. Jiang H. Meng Y. Wang H. Li Y. (2023). Flight training changes the brain functional pattern in cadets. *Front. Neurosci.* 17:1120628. 10.3389/fnins.2023.1120628 37025375 PMC10070807

[B19] Cole M. W. Reynolds J. R. Power J. D. Repovs G. Anticevic A. Braver T. S. (2013). Multi-task connectivity reveals flexible hubs for adaptive task control. *Nat. Neurosci.* 16 1348–1355. 10.1038/nn.3470 23892552 PMC3758404

[B20] Dahlstrom N. Nahlinder S. (2009). Mental workload in aircraft and simulator during basic civil aviation training. *Int. J. Aviat. Psychol.* 19 309–325. 10.1080/10508410903187547

[B21] Deco G. Jirsa V. K. McIntosh A. R. (2011). Emerging concepts for the dynamical organization of resting-state activity in the brain. *Nat. Rev. Neurosci.* 12 43–56. 10.1038/nrn2961 21170073

[B22] Egner T. Siqi-Liu A. (2024). Insights into control over cognitive flexibility from studies of task-switching. *Curr. Opin. Behav. Sci.* 55:101342. 10.1016/j.cobeha.2023.101342 38186744 PMC10769152

[B23] Evrard H. C. (2019). The Organization of the primate insular cortex. *Front. Neuroanat.* 13:43. 10.3389/fnana.2019.00043 31133822 PMC6517547

[B24] Fan L. Li H. Zhuo J. Zhang Y. Wang J. Chen L. (2016). The human brainnetome atlas: A new brain atlas based on connectional architecture. *Cereb. Cortex* 26 3508–3526. 10.1093/cercor/bhw157 27230218 PMC4961028

[B25] Funahashi S. (2017). Working Memory in the prefrontal cortex. *Brain Sci.* 7:49. 10.3390/brainsci7050049 28448453 PMC5447931

[B26] Gao K. He H. Lu B. Xie Q. Lu J. Yao D. (2024). Discrepant changes in structure–function coupling in dancers and musicians. *Cereb. Cortex* 34:bhae068. 10.1093/cercor/bhae068 38489785

[B27] Griffa A. Amico E. Liégeois R. Van De Ville D. Preti M. G. (2022). Brain structure-function coupling provides signatures for task decoding and individual fingerprinting. *Neuroimage* 250:118970. 10.1016/j.neuroimage.2022.118970 35124226

[B28] Grill-Spector K. Malach R. (2004). The human visual cortex. *Annu. Rev. Neurosci.* 27 649–677. 10.1146/annurev.neuro.27.070203.144220 15217346

[B29] Honey C. J. Sporns O. Cammoun L. Gigandet X. Thiran J. P. Meuli R. (2009). Predicting human resting-state functional connectivity from structural connectivity. *Proc. Natl. Acad. Sci. U.S.A.* 106 2035–2040. 10.1073/pnas.0811168106 19188601 PMC2634800

[B30] Huang W. Bolton T. A. W. Medaglia J. D. Bassett D. S. Ribeiro A. Van De Ville D. (2018). A Graph signal processing perspective on functional brain imaging. *Proc. IEEE* 106 868–885. 10.1109/JPROC.2018.2798928

[B31] Ji M. You X. Lan J. Yang S. (2011). The impact of risk tolerance, risk perception and hazardous attitude on safety operation among airline pilots in China. *Saf. Sci.* 49 1412–1420. 10.1016/j.ssci.2011.06.007

[B32] Jillings S. Pechenkova E. Tomilovskaya E. Rukavishnikov I. Jeurissen B. Van Ombergen A. (2023). Prolonged microgravity induces reversible and persistent changes on human cerebral connectivity. *Commun. Biol.* 6:46. 10.1038/s42003-022-04382-w 36639420 PMC9839680

[B33] Kharoufah H. Murray J. Baxter G. Wild G. (2018). A review of human factors causations in commercial air transport accidents and incidents: From to 2000–2016. *Prog. Aeros. Sci.* 99 1–13. 10.1016/j.paerosci.2018.03.002

[B34] Lee H.-B. Park J.-W. (2016). Comparative study on hazardous attitudes and safe operational behavior in airline pilots. *J. Air Trans. Manag.* 54 70–79. 10.1016/j.jairtraman.2016.03.024

[B35] Li Y. Seger C. Chen Q. Mo L. (2020). Left inferior frontal gyrus integrates multisensory information in category learning. *Cereb. Cortex* 30 4410–4423. 10.1093/cercor/bhaa029 32133488

[B36] Lin Y.-H. Young I. M. Conner A. K. Glenn C. A. Chakraborty A. R. Nix C. E. (2020). Anatomy and white matter connections of the inferior temporal gyrus. *World Neurosurg.* 143 e656–e666. 10.1016/j.wneu.2020.08.058 32798785

[B37] May A. (2011). Experience-dependent structural plasticity in the adult human brain. *Trends Cogn. Sci.* 15 475–482. 10.1016/j.tics.2011.08.002 21906988

[B38] Mišić B. Betzel R. F. De Reus M. A. Van Den Heuvel M. P. Berman M. G. McIntosh A. R. (2016). Network-level structure-function relationships in human neocortex. *Cereb. Cortex* 26 3285–3296. 10.1093/cercor/bhw089 27102654 PMC4898678

[B39] Mišić B. Betzel R. F. Griffa A. De Reus M. A. He Y. Zuo X.-N. (2018). Network-Based asymmetry of the human auditory system. *Cereb. Cortex* 28 2655–2664. 10.1093/cercor/bhy101 29722805 PMC5998951

[B40] Palejwala A. H. (2021). Anatomy and White matter connections of the lingual gyrus and cuneus. *World Neurosurg.* 151 e426–e437. 10.1016/j.wneu.2021.04.050 33894399

[B41] Parker J. G. Zalusky E. J. Kirbas C. (2014). Functional MRI mapping of visual function and selective attention for performance assessment and presurgical planning using conjunctive visual search. *Brain Behav.* 4 227–237. 10.1002/brb3.213 24683515 PMC3967538

[B42] Preti M. G. Van De Ville D. (2019). Decoupling of brain function from structure reveals regional behavioral specialization in humans. *Nat. Commun.* 10:4747. 10.1038/s41467-019-12765-7 31628329 PMC6800438

[B43] Qiu C. Zhao C. Hu G. Zhang Y. Zhu Y. Wu X. (2021). Brain structural plasticity in visual and sensorimotor areas of airline pilots: A voxel-based morphometric study. *Behav. Brain Res.* 411:113377. 10.1016/j.bbr.2021.113377 34023308

[B44] Qiu Y. Wu Y. Liu R. Wang J. Huang H. Huang R. (2019). Representation of human spatial navigation responding to input spatial information and output navigational strategies: An ALE meta-analysis. *Neurosci. Biobehav. Rev.* 103 60–72. 10.1016/j.neubiorev.2019.06.012 31201830

[B45] Radstake W. E. Jillings S. Laureys S. Demertzi A. Sunaert S. Van Ombergen A. (2023). Neuroplasticity in F16 fighter jet pilots. *Front. Physiol.* 14:1082166. 10.3389/fphys.2023.1082166 36875024 PMC9974643

[B46] Rajesh A. Seider N. A. Newbold D. J. Adeyemo B. Marek S. Greene D. J. (2024). Structure–function coupling in highly sampled individual brains. *Cereb. Cortex* 34:bhae361. 10.1093/cercor/bhae361 39277800 PMC12098013

[B47] Rivas-Fernández M. Á Varela-López B. Cid-Fernández S. Galdo-Álvarez S. (2021). Functional Activation and connectivity of the left inferior frontal gyrus during lexical and phonological retrieval. *Symmetry* 13:1655. 10.3390/sym13091655

[B48] Salgado-Pineda P. Baeza I. Pérez-Gómez M. Vendrell P. Junqué C. Bargalló N. (2003). Sustained attention impairment correlates to gray matter decreases in first episode neuroleptic-naive schizophrenic patients. *Neuroimage* 19 365–375. 10.1016/S1053-8119(03)00094-6 12814586

[B49] Seguin C. Van Den Heuvel M. P. Zalesky A. (2018). Navigation of brain networks. *Proc. Natl. Acad. Sci. U.S.A.* 115 6297–6302. 10.1073/pnas.1801351115 29848631 PMC6004443

[B50] Seyfzadehdarabad F. Sadeghi-Firoozabadi V. Shokri O. Bagheri M. Sadeghi Firoozabadi A. (2023). Cognitive correlates of maritime pilots’ human errors. *Saf. Sci.* 165:106196. 10.1016/j.ssci.2023.106196

[B51] Shomstein S. Lee J. Behrmann M. (2010). Top-down and bottom-up attentional guidance: Investigating the role of the dorsal and ventral parietal cortices. *Exp. Brain Res.* 206 197–208. 10.1007/s00221-010-2326-z 20571784 PMC5728384

[B52] Sladky R. Stepniczka I. Boland E. Tik M. Lamm C. Hoffmann A. (2016). Neurobiological differences in mental rotation and instrument interpretation in airline pilots. *Sci. Rep.* 6:28104. 10.1038/srep28104 27323913 PMC4914984

[B53] Stiso J. Bassett D. S. (2018). Spatial embedding imposes constraints on neuronal network architectures. *Trends Cogn. Sci.* 22 1127–1142. 10.1016/j.tics.2018.09.007 30449318

[B54] Uddin L. Q. (2021). Cognitive and behavioural flexibility: Neural mechanisms and clinical considerations. *Nat. Rev. Neurosci.* 22 167–179. 10.1038/s41583-021-00428-w 33536614 PMC7856857

[B55] Vandenberghe R. Molenberghs P. Gillebert C. R. (2012). Spatial attention deficits in humans: The critical role of superior compared to inferior parietal lesions. *Neuropsychologia* 50 1092–1103. 10.1016/j.neuropsychologia.2011.12.016 22266260

[B56] Vázquez-Rodríguez B. Suárez L. E. Markello R. D. Shafiei G. Paquola C. Hagmann P. (2019). Gradients of structure–function tethering across neocortex. *Proc. Natl. Acad. Sci. U.S.A.* 116 21219–21227. 10.1073/pnas.1903403116 31570622 PMC6800358

[B57] Wang G. Jiang N. Liu T. Wang L. Suo D. Chen D. (2024). Using unsupervised capsule neural network reveal spatial representations in the human brain. *Hum. Brain Mapp.* 45:e26573. 10.1002/hbm.26573 38544416 PMC10973701

[B58] Wang H. Pan T. Si H. Li Y. Jiang N. (2020). Research on influencing factor selection of pilot’s intention. *Int. J. Aeros. Eng.* 2020 1–13. 10.1155/2020/4294538

[B59] Wang J. Yang Y. Zhao X. Zuo Z. Tan L.-H. (2020). Evolutional and developmental anatomical architecture of the left inferior frontal gyrus. *Neuroimage* 222:117268. 10.1016/j.neuroimage.2020.117268 32818615

[B60] Wang L. Kakigi R. Hoshiyama M. (2001). Neural activities during wisconsin card sorting test — MEG observation. *Cogn. Brain Res.* 12 19–31. 10.1016/S0926-6410(01)00022-2 11489605

[B61] Wang Y. (2025). Neural representation of response inhibition and attentional capture in the right inferior frontal gyrus. *Eur. J Neurosci.* 61:e70048. 10.1111/ejn.70048 40029550

[B62] Wu D. Fan L. Song M. Wang H. Chu C. Yu S. (2020). Hierarchy of connectivity–function relationship of the human cortex revealed through predicting activity across functional domains. *Cereb. Cortex* 30 4607–4616. 10.1093/cercor/bhaa063 32186724 PMC8248587

[B63] Xu K. Liu R. Chen X. Yang Y. Wang Q. (2023). Brain structure variability study in pilots based on VBM. *PLoS One* 18:e0276957. 10.1371/journal.pone.0276957 36706169 PMC9882760

[B64] Ye L. Ba L. Yan D. (2025). A study of dynamic functional connectivity changes in flight trainees based on a triple network model. *Sci. Rep.* 15:7828. 10.1038/s41598-025-89023-y 40050304 PMC11885617

[B65] Yeo B. T. T. Krienen F. M. Eickhoff S. B. Yaakub S. N. Fox P. T. Buckner R. L. (2015). Functional specialization and flexibility in human association cortex. *Cereb. Cortex* 25 3654–3672. 10.1093/cercor/bhu217 25249407 PMC4598819

