# Correction: Neuroadaptive changes in brain structural–functional coupling among pilots

**DOI:** 10.3389/fnins.2026.1807870

**Published:** 2026-02-25

**Authors:** Xi Chen, Qingbin Meng, Qingsong Song, Peiran Xu, Shicong Zhang, Donglin Huang, Qi Chu, Jiamin Fan, Cheng Luo, Xiuyi Li

**Affiliations:** 1 Institute of Flight Technology, Civil Aviation Flight University of China, Guanghan, Sichuan, China; 2 Aviation Health Department, Southwest Regional Administration of Civil Aviation Administration of China, Chengdu, China; 3 Hospital of Civil Aviation Flight University of China, Civil Aviation Flight University of China, Guanghan, Sichuan, China; 4 Key Laboratory for Neuroinformation of Ministry of Education, School of Life Sciences and Technology, University of Electronic Science and Technology of China, Chengdu, China; 5 CAAC Academy, Civil Aviation Flight University of China, Guanghan, China

**Keywords:** structural-functional coupling, flying experience, neuroplasticity, function connectivity, graph signal processing

An incorrect number was provided for the National Nature Science Foundation of China (No. U2033209). The correct number is No. U2133209.

The original version of this article has been updated.

