# Supplementary material for: Neuroadaptive changes in brain structural–functional coupling among pilots
Source: Front Neurosci. 2025 Jul 24;19:1608739. doi: 10.3389/fnins.2025.1608739 (PMC12329225; doi:10.3389/fnins.2025.1608739)
Supplement: Supplementary file 1 [file Table_1.docx]

Supplementary Material

# Supplementary Tables

**Table S1.** Differential brain region information

|  |  |  |  | **MNI** |  |  |
| --- | --- | --- | --- | --- | --- | --- |
| **Hemisphere** | **Number** | **Anatomy area** | **x** | **y** | **z** | **T-value** |
| Left | L_MFG_7_2 | Middle Frontal Gyrus^*^ | -42 | 13 | 36 | 2.228405 |
| Right | R_MFG_7_7 | Middle Frontal Gyrus^*^ | 25 | 61 | -4 | 2.20445 |
| Right | R_IFG_6_3 | Inferior Frontal Gyrus^*^ | 54 | 24 | 12 | -2.15118 |
| Left | L_IFG_6_6 | Inferior Frontal Gyrus^*^ | -52 | 13 | 6 | -2.0335 |
| Left | L_PrG_6_1 | Precentral Gyrus^*^ | -49 | -8 | 39 | 2.427638 |
| Left | L_PrG_6_4 | Precentral Gyrus^*^ | -13 | -20 | 73 | -2.02529 |
| Left | L_ITG_7_2 | Inferior Temporal Gyrus^*^ | -51 | -57 | -15 | 2.110466 |
| Left | L_ITG_7_5 | Inferior Temporal Gyrus^*^ | -55 | -60 | -6 | 2.352456 |
| Right | R_ITG_7_5 | Inferior Temporal Gyrus^*^ | 54 | -57 | -8 | 2.020918 |
| Left | L_ITG_7_6 | Inferior Temporal Gyrus^*^ | -59 | -42 | -16 | 2.018789 |
| Left | L_pSTS_2_1 | posterior Superior Temporal Sulcus^**^ | -54 | -40 | 4 | 2.805622 |
| Right | R_SPL_5_3 | Superior Parietal Lobule^*^ | 35 | -42 | 54 | 2.115428 |
| Right | R_IPL_6_5 | Inferior Parietal Lobule^*^ | 53 | -54 | 25 | 2.348054 |
| Left | L_INS_6_4 | Insula^*^ | -38 | -4 | -9 | -2.15366 |
| Left | L_MVOcC_5_2 | Medial Visual Occipital Cortex^*^ | -5 | -81 | 10 | 2.484303 |
| Right | R_BG_6_4 | Basal Ganglia^*^ | 22 | 8 | -1 | 2.012513 |

**: p < 0.01 (permutation test), *: p < 0.05 (permutation test).
